# Absolute Antioxidant Activity of Five Phenol-Rich Essential Oils

**DOI:** 10.3390/molecules26175237

**Published:** 2021-08-29

**Authors:** Yafang Guo, Romeo Pizzol, Simone Gabbanini, Andrea Baschieri, Riccardo Amorati, Luca Valgimigli

**Affiliations:** 1 Department of Chemistry “G. Ciamician”, University of Bologna, Via S. Giacomo 11, 40126 Bologna, Italy; yafang.guo2@unibo.it (Y.G.); romeo.pizzol@studio.unito.it (R.P.); riccardo.amorati@unibo.it (R.A.); 2 Research & Development—BeC s.r.l., Via C. Monteverdi 49, 47122 Forlì, Italy; laboratorio@bec-natura.com; 3 The Institute of Organic Synthesis and Photoreactivity, Consiglio Nazionale delle Ricerche (CNR), Via P. Gobetti 101, 40129 Bologna, Italy; andrea.baschieri@isof.cnr.it

**Keywords:** essential oils, antioxidant, GC-MS, thyme, oregano, savory, clove, cinnamon, peroxyl radicals

## Abstract

Essential oils (EOs) have promising antioxidant activities which are gaining interest as natural alternatives to synthetic antioxidants in the food and cosmetic industries. However, quantitative data on chain-breaking activity and on the kinetics of peroxyl radical trapping are missing. Five phenol-rich EOs were analyzed by GC-MS and studied by oxygen-uptake kinetics in inhibited controlled autoxidations of reference substrates (cumene and squalene). Terpene-rich *Thymus vulgaris* (thymol 4%; carvacrol 33.9%), *Origanum vulgare*, (thymol 0.4%; carvacrol 66.2%) and *Satureja hortensis*, (thymol 1.7%; carvacrol 46.6%), had apparent *k*_inh_ (30 °C, PhCl) of (1.5 ± 0.3) × 10^4^, (1.3 ± 0.1) × 10^4^ and (1.1 ± 0.3) × 10^4^ M^−1^s^−1^, respectively, while phenylpropanoid-rich *Eugenia caryophyllus* (eugenol 80.8%) and *Cinnamomum zeylanicum*, (eugenol 81.4%) showed apparent *k*_inh_ (30 °C, PhCl) of (5.0 ± 0.1) × 10^3^ and (4.9 ± 0.3) × 10^3^ M^−1^s^−1^, respectively. All EOs already granted good antioxidant protection of cumene at a concentration of 1 ppm (1 mg/L), the duration being proportional to their phenolic content, which dictated their antioxidant behavior. They also afforded excellent protection of squalene after adjusting their concentration (100 mg/L) to account for the much higher oxidizability of this substrate. All investigated EOs had *k*_inh_ comparable to synthetic butylated hydroxytoluene (BHT) were are eligible to replace it in the protection of food or cosmetic products.

## 1. Introduction

Among the many properties that are often attributed to plant essential oils (EOs), the antioxidant property certainly stands out [1]. When paired with the antimicrobial activity, which has been extensively documented for many essential oils in recent and less recent literature [2,3,4], the additional antioxidant activity makes those natural materials extremely attractive as multi-functional preservatives, e.g., for food products, able to control spoilage caused both by microbial metabolism and by air oxidation [3,5]. In recent years, consumer choice has progressively oriented food technologists toward more natural and (expectedly) safer alternatives to synthetic additives, which has boosted the interest in plant-derived bioactives, including essential oils [1,2,3,5,6].

On the other hand, essential oils have long-standing traditions of use in several field, ranging from cosmetics to medicine, from the preservation of artistic heritage to veterinary and agriculture applications, and many such uses are based on their purported antioxidant activity [7,8,9,10,11,12,13,14]. Not surprisingly, therefore, several investigations on the antioxidant activity of EOs have appeared in the scientific literature. However, the majority of such studies have only focused on qualitative assessment or have relied on popular assays based on single-point assessment of their reducing ability toward some oxidizing species (e.g., Fe^3+^) or persistent artificial radicals (e.g., DPPH or ABTS) [5]. While these assays can shed light on a potential activity, they cannot provide quantitative absolute descriptors of the antioxidant activity [15]—namely, the kinetic constants for trapping peroxyl radicals and breaking the oxidative chain-reaction [16,17,18]—and their results depend on the experimental settings, making it difficult to compare or rationalize the data [5,15,18]. In other cases, studies were based on real-life applications, such as on tests of food spoilage [19,20,21], which are certainly relevant but often cannot distinguish what is caused by microorganisms and what is caused by air oxidation [5]. These limits have already been discussed in detail [5,15,16,17,18]; however, a clear problem arises when trying to overcome them, in that, at variance with single-molecule antioxidants, essential oils are complex mixtures of a few to dozens of components, whose individual contribution to the properties of the whole oil is often unknown.

In this study, we are presenting and testing a simplified approach to deal with this complexity, at least in the case of phenol-rich EOs, which are abundant in nature. Five essential oils from different botanical sources, of very common use in the food industry as well as in the cosmetic and other industries [5], were chosen as representative prototypes of phenol-rich EOs, namely red thyme (*Thymus vulgaris*, L.), oregano (*Origanum vulgare*, L.), savory (*Satureja hortensis*, L.), which is rich in terpenic phenols, clove bud (*Eugenia caryophyllus*, Spreng. or *Syzygium aromaticum*, L.), and cinnamon leaves (*Cinnamomum zeylanicum*, Blume), which is rich in phenylpropanoids. Since there is major variability in the composition of EOs, even within the same botanical species, due to chemotype, climate, soil composition, cultivation technique, plant collection time, and extraction method [22,23,24], we subjected our specimens to composition analysis prior to studying their reactivity with peroxyl radicals by means of inhibited autoxidation studies [25,26,27], which represents the gold standard in antioxidant testing [15,17,18].

To our knowledge, this is the first report of the absolute kinetics of the trapping of peroxyl radicals by these essential oils. The results support their reputation as good antioxidants and provide quantitative grounds for their eligibility as replacement for synthetic antioxidants in specific applications.

## 2. Results and Discussion

### 2.1. Phenolic Compositions of the Essential Oils

The composition of the EO specimens used in this study was determined by CG-MS analysis. Identification of the components was based on Kovat’s Index [28], and on matching the mass spectrum with the NIST (National Institute of Standards and Technology, USA) library [29] and with an internal (self-built) spectral library for essential oils. Chromatograms and details for components’ assignment are provided as Supplementary Material, while results are summarized in Table 1. To simplify the assignment, components with peak area <0.1% were not investigated, since our focus was on the antioxidant activity and no significant contribution can reasonably be expected from such minor components under our experimental design, based on testing the essential oils at very low concentrations (see Section 2.2). To gain a more accurate measurement of the relative abundance of components in each oil in the absence of calibration with authentic standards of individual components, we turned to the flame ionization detector (FID) method, since GC-FID normally offers better accuracy and better linear range as compared to GC-MS under these conditions [30]. Although this approach does not offer the same accuracy as true calibration [30], previous experience under identical instrumental settings indicated a variation ≤10% of the relative response factor (RRF expressed as mass ratio) among similar EO components, which was judged sufficient to the scope of our investigation. Therefore, following the identification of components by GC-MS, samples were re-analyzed under identical settings in GC-FID, taking advantage of a combined instrument (see Section 3.2).

**Figure 1:**
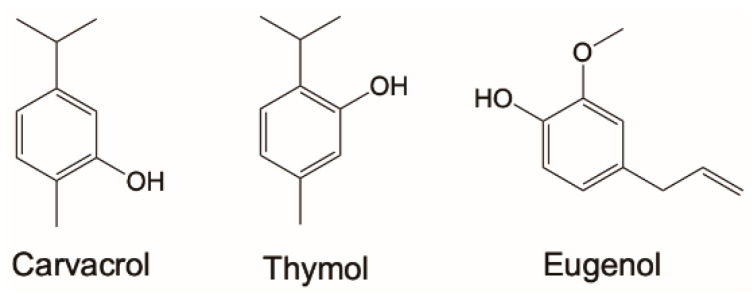
Phenolic components found in the investigated EOs.

Red thyme allowed for the identification of seven components, accounting for 99.8% of the total peak area in the chromatogram, which included two phenolic compounds (Figure 1): thymol (4%) and carvacrol (33.9%) summing up to 37.9% of total phenolics. In oregano, carvacrol was the main component (69.2%) and thymol was the only other phenolic component we found, albeit at much lower level (0.4%) summing up to 69.6% of total phenolics. In savory, we identified 16 components which accounted for 97.1% of total peak area; among them again thymol (1.7%) and carvacrol (46.6%) were the only phenolic components, summing up to 48.3%. The predominant content of clove bud oil was based on phenylpropanoids. Among the four identified components, which accounted for 99.9% of total peak area, eugenol (Figure 1) was the only phenolic component, representing 80.8% of the oil. Cinnamon leaves EO had a more complex structure, with 11 identified components representing 100% of the chromatogram area; however, eugenol was the only phenolic component, accounting for 81.4% the essential oil.

Overall, the composition of the investigated EOs was in line with the expected components’ range and levels [3] and highlights the presence of only three phenolic components (Figure 1), which represented 38–81% of the investigated oils, promising a relevant chain-breaking antioxidant activity.

### 2.2. Autoxidation of Reference Substrates Inhibited by Essential Oils

We tested the EOs in the controlled autoxidation of a kinetically characterized oxidizable substrate, which is the best-established approach for quantitative testing [16,18,31]. This test was based on monitoring the progress of the autoxidation of a reference substrate by measuring the rate of oxygen consumption in the presence and absence of the test antioxidant. When autoxidation is initiated at a constant rate *R*_i_, controlled by the thermal decomposition of an azo-iniziator like 2,2′-azobis(isobutyronitrile) (AIBN), the process can be described by Equations (1)–(6),

R-N=N-R → 2 R● + N_2_ (1)

R● + O_2_ → ROO● (2)

RH + ROO● → R● + ROOH(3)

2 ROO● → non radical products(4)

AH + ROO● → A● + ROOH(5)

A● + ROO● → ROOA(6)

where *R*_i_ is coincident with the rate of reaction 1. Equations (1)–(4) represent the autoxidation of substrate RH in the absence of antioxidants, while Equations (5) and (6) represent chain-breaking inhibition by antioxidant AH, e.g., a phenolic compound able to transfer the phenolic O-H to a chain-carrying peroxyl radical ROO●. Upon reaction with peroxyl radicals (Equation (5)) phenolic antioxidants form stabilized radicals that will normally not propagate the chain, but instead “wait in solution” to trap a second peroxyl radical (Equation (6)). For this reason, phenols are known to have a stoichiometric factor of *n* = 2, i.e., each molecule of antioxidant would trap two peroxyl radicals. While this affects primarily the duration of protection, the efficacy of protection depends primarily on *k*_inh_, the inhibition rate constant, which is coincident with the rate constant of reaction 5, being the rate-limiting step of inhibition.

**Figure 2:**
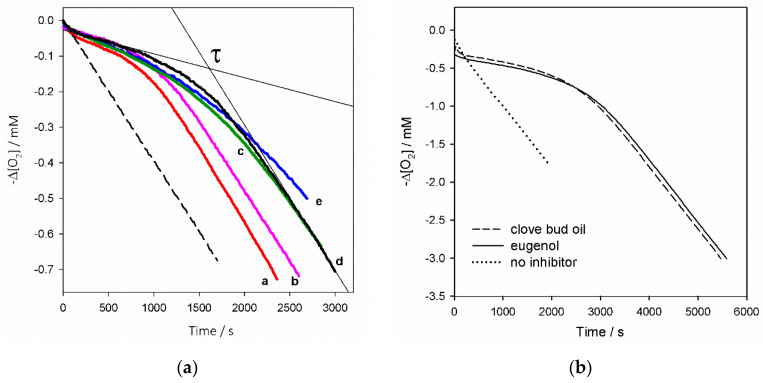
Oxygen consumption during the autoxidation of cumene (3.6 M) initiated by AIBN (0.05 M) in PhCl at 30 °C (panel **a**) without inhibitors (dashed) or in the presence of EOs (1 mg/L): red thyme (a), savory (b), clove bud (c), oregano (d), cinnamon leaves (e); where inhibition time τ is determined at the cross-point between inhibited and uninhibited tracts as illustrated for plot (d); and (panel **b**) without inhibitors (dotted), or in the presence of 1 mg/L eugenol (full), or clove bud oil (1.25 mg/L) dosed to provide 1 mg/L eugenol (dashed).

We first tested the five EOs for their ability to inhibit the autoxidation of cumene in chlorobenzene (PhCl) solution, which is among the best-established reference oxidizable substrates [16,32]. All EOs were tested at the very low concentration of 1 mg/L (1 ppm, 0.0001% *w*/*v*), in order to deconvolute the antioxidant behavior and distinguish chain-breaking inhibition (described by Equations (5) and (6)) from other mechanisms like termination-enhancing, previously observed for some EO components [33], which becomes relevant only at rather high concentrations of the antioxidant. The results summarized in Figure 2a show that all tested EOs afforded good protection, giving neat inhibition of the autoxidation for a time τ, which lasted until the antioxidants were consumed, then oxygen consumption restarted at uninhibited rate (see Figure 2a, plot **d**). During the inhibited period, the rate of oxygen consumption is described by Equation (7), where *n* is the stoichiometric factor (*n* = 2 for phenols, see above) and *k*_p_ is the rate constant of oxidative chain propagation, coincident with the rate constant of reaction 3—a specific property of each oxidizable substrate that determines its tendency to oxidize. Since *k*_p_ is known for cumene (0.34 M^−1^s^−1^ at 30 °C) [16], and *R*_ì_ is set in preliminary experiments, fitting the oxygen uptake plots with Equation (7) would afford *k*_inh_, i.e., the absolute rate constant for the reaction of the antioxidant with peroxyl radicals (Equation (5)), provided the molar concentration of the substrate [RH] and of the antioxidant [AH] are known. Unlike for single-molecule antioxidants, this last provision is not obvious for complex mixtures like EOs. In this study, to make the kinetic analysis tractable, we hypothesized that, to a first approximation, the antioxidant activity arises from the phenolic components in each EOs, all having an expected value of *n* = 2. Under this hypothesis, the initial effective concentration [AH]_0_ can be simply obtained from the duration (τ) of the inhibited period, according to Equation (8).

(7) $$-\frac{d\left[O2\right]}{dt}=\frac{k_{p}\left[\mathrm{RH}\right]R_{i}}{nk_{\mathrm{inh}}\left[\mathrm{AH}\right]}+R_{i}$$

(8) $$R_{i}=\frac{n{\left[\mathrm{AH}\right]}_{0}}{\tau }$$

The calculated value of [AH] was then used in Equation (7) to afford the absolute apparent values of *k*_inh_ for each tested EO. The results are summarized in Table 2.

Values of *k*_inh_ for peroxyl radical trapping are disclosed for the first time for the investigated essential oils. Results indicate a clustering of the EOs containing thymol and carvacrol around similar *k*_inh_ values (1.3 × 10^4^ to 1.5 × 10^4^ M^−1^s^−1^), while the two EOs rich in eugenol showed a somewhat lower value around 5 × 10^3^ M^−1^s^−1^.

These last results can be matched with the previously reported value of *k*_inh_ for pure eugenol (4.8 × 10^3^ M^−1^s^−1^ [34]) to which they are superimposable within experimental error. Although there is no value in the literature for the *k*_inh_ of thymol and carvacrol, other structurally related phenols can be taken as reference, e.g., 2,6-dimethylphenol (*k*_inh_ = 1.5 × 10^4^ M^−1^s^−1^, 30 °C, PhCl [16]), bearing two alkyl substituents on the ring, albeit one in a different position. Since alkyl substituents in the ortho position are known to express higher electron donating contributions than in meta, but would also cause higher steric hindrance and the two factors affect the reactivity in opposite directions [16], to a first approximation we can expect both thymol and carvacrol to have *k*_inh_ ~ 1.5 × 10^4^ M^−1^s^−1^. This value matches the *k*_inh_ value measured here for EOs containing the two terpenic phenols. Therefore, it appears that the *k*_inh_ for EOs, i.e., their efficiency in trapping peroxyl radicals, largely copies that of the prevailing phenolic component. On the other hand, the duration of antioxidant protection was significantly higher for oregano, clove bud, and cinnamon leaves, as compared to the remaining two oils, as is clearly visible in Figure 2a. This translates into higher values of effective or apparent antioxidant concentration [AH]_app_ in the autoxidizing mixture (as determined by Equation (8)), despite the fact that all EOs were used at the same concentration (1 mg/L).

It is interesting to compare the values of [AH]_app_ measured in autoxidations with the estimated molar concentration of EOs phenolic components in the autoxidizing mixture, which were obtained from analysis of the EOs. The estimated total phenolic concentration reported in Table 2 matches the measured effective antioxidant concentration within experimental error, with only cinnamon showing an [AH]_app_ value slightly lower that the estimated content of eugenol (4.4 ± 0.4 µM vs. 5.0 µM). This minor difference, however, is perfectly justified by the somewhat lower accuracy in determining τ (see Section 3.3) and by the simplicity of our analytical approach, which assumes equal RRF for all EO components, and does not change the main finding: the antioxidant activity of phenol-rich EOs is dictated by their phenolic content, both in terms of efficiency in peroxyl radical trapping and of the duration of the antioxidant protection. As a proof of concept, we performed matched autoxidations of cumene inhibited either by 1 mg/L of authentic eugenol or by clove bud EO dosed so as to bring the same amount of eugenol to the mixture. As can be seen in Figure 2b, the oxygen consumption traces are largely superimposable, confirming our initial hypothesis that the antioxidant activity of phenol-rich EOs arises nearly entirely from their phenolic components.

While this might seem to be a reasonable finding, it is at odds with some previous reports in the field. For instance, a recent study on the antioxidant activity of cinnamon EO by DPPH and H_2_O_2_ scavenging assays attributed the property to α-pinene and sesquiterpenes [35], yet Kaur et al., using the DPPH assay, found that clove bud oil had higher antioxidant activity than eugenol isolated from the oil [36]. Clearly, the major difference between our inhibited autoxidation studies and those assays plays a role in such different outcomes.

In order to confirm our results on a different substrate that would be more challenging to protect and more representative of highly oxidizable food products or ingredients used in cosmetics and pharmaceuticals, we investigated the inhibited autoxidation of squalene. This ubiquitous polyunsaturated triterpenic hydrocarbon composes the unsaponifiable fraction of vegetable oils, particularly of olive, from which it is obtained [37], and it has immune-stimulating bioactivity that makes it interesting as a diet supplement [38] and pharmaceutical ingredient (e.g., in the composition of vaccines) [39], besides being very popular in the cosmetic industry as skin emollient and protectant [37]. Its kinetics of autoxidation was recently characterized in our group highlighting a *k*_p_ value of 68 M^−1^s^−1^ (30 °C) [40], i.e., 200-fold faster than cumene, and even faster than linoleic acid, which often taken as prototype for highly oxidizable polyunsaturated lipids [40], which makes its protection quite challenging.

**Figure 3:**
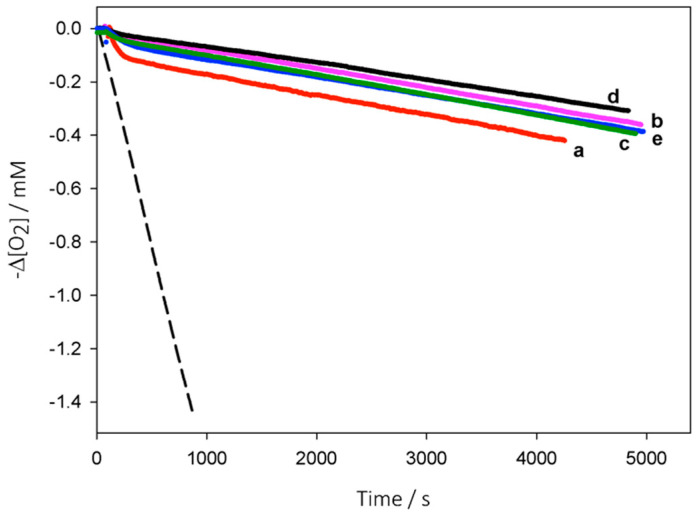
Oxygen consumption during the autoxidation of squalene (1.04 M) initiated by AIBN (0.05 M) in PhCl at 30 °C without inhibitors (dashed) or in the presence of EOs (100 mg/L): red thyme (a), savory (b), clove bud (c), oregano (d), and cinnamon leaves (e).

When we tested our EOs with squalene at the concentration of 1 mg/L, no protection was detectable and only on increasing the concentration some slowing down of the oxygen consumption was observed, albeit without a clear inhibition period (see Figure S6 in the Supplementary Material). However, when we raised the concentration to 100 mg/L, to (partly) compensate for the much higher *k*_p_ of squalene, excellent protection was granted by all the EOs, as displayed in Figure 3. It should be noted that the increased concentration of EOs in this study corresponds to 0.02% (*w*/*v*) compared to the oxidizable substrate, a value compatible with any technological application and much lower than that typically used for antioxidants in foods or cosmetics (0.1%).

Analysis of the oxygen consumption traces (Figure 3) was performed better with a different mathematical treatment due to the absence of a visible inhibited period (see Section 3.3), and results were compared to those obtained with cumene in Table 2. As expected, the *k*_inh_ values measured with squalene were very close to those obtained with cumene, when the experimental error and the difference in the analysis method are considered.

It is interesting to note that those values (average of cumene and squalene) are of the same magnitude as the *k*_inh_ of the very common antioxidant BHT (butylated hydroxyanisole or 2,6-di-*tert*-butyl-4-methylphenol), reported as 1.0 × 10^4^ M^−1^s^−1^ (30 °C) [41]; therefore, the investigated EOs afford equivalent antioxidant protection.

### 2.3. Study Limitations and Future Directions

GC-FID analysis with actual calibration, or the use of relative response factors (RRF) calculated for each component brings to higher analytical accuracy [42]. However, our estimation of phenolic EO components (Table 2) from semiquantitative GC-FID, assuming the same mass-based RRF for all components is justified by the proportionality of the FID response factor to the number of carbons in the analyte and was sufficient for the purpose of this investigation under our settings.

One distinctive advantage to our approach, which investigates the chain-braking antioxidant activity of EOs at very low concentration, is that it allows us to deconvolute this most relevant mechanism from artifacts due to the oxidizability of many non-phenolic components, which (at higher concentration) would reduce radicals bringing to misleading results, as was shown to occur with persistent radicals like DPPH, or oxidizing species like Fe^3+^ or phosphomolibdate used in popular assays, [5]. However, other mechanisms of antioxidant activity by EOs, such as the termination-enhancing mechanism, have been shown to occur at higher concentration due to the chemistry of some non-phenolic components [43]. Testing phenol-poor EOs under different settings would complement our current approach and provide a more complete picture.

A second advantage of the approach used herein is that the autoxidation kinetics were performed at low temperature, thus reducing the loss of volatile components through evaporation. This is clearly an advantage if compared to accelerated oxidation methods based on bubbling a stream of air in heated lipid samples (e.g., lard), in which volatile antioxidant evaporation may explain the limited efficacy of essential oil components [44].

For phenol-rich EOs, our current results offer a relatively simple yet highly reliable means for predicting and rationalizing antioxidant properties based on phenolic composition. However, the approach only tested a limited number of EOs, all containing a limited number of phenolic components. Clearly it would be useful to validate it on a broader and more diverse set of EOs, which we plan to pursue in future work.

## 3. Materials and Methods

### 3.1. Materials

All chemicals and solvents were commercially available (Aldrich-Fluka-Sigma-Merck, Milan, Italy, unless otherwise noted). 2,2′-Azobis(isobutyronitrile) (AIBN) was recrystallized from methanol. 2,2,5,7,8-Pentamethyl-6-chromanol (PMHC) was recrystallized from hexane. Squalene (≥98%) and cumene (≥98%) were percolated twice through activated basic alumina and once through silica to remove impurities and traces of hydroperoxides [30,42]. Eugenol (natural, ≥98%) was used as received. Essential oils of red thyme (*T. vulgaris*, L.), oregano (*O. vulgare*, L.), and savory (*S. hortensis*, L.) were purchased from Maraschi & Quirici s.p.a. (Riva Presso Chieri, Italy); the EOs of clove bud (*E. caryophyllus*, Spreng.) and cinnamon leaves (*C. zeylanicum*, Blume) were from Muller & Koster s.p.a. (Milano, Italy). All solutions were in chlorobenzene (99.9% HPLC grade).

### 3.2. GC-MS and GC-FID Analysis of the EOs

GC–MS analysis was carried out on a Star 3400 CX gas chromatograph (Varian, Palo Alto, CA, USA) equipped with a Saturn 2000 ion-trap mass spectrometer detector (Varian), and with a flame ionization detector (FID; H_2_/Air), mounting 2 split/splitless 1078 Universal Capillary Injectors (Varian), each leading to a capillary column (Zebron ZB-5, 5% phenyl-95% dimethyl-polysiloxane, 30m × 0.25mm × 0.25µm) from Phenomenex (Torrance, CA, USA.), each connected to one of the two detectors (MS or FID). The instrument was also equipped with a CombiPAL 2-axis autosampler (CTC Analytics, Zwingen, Switzerland). The carrier was helium at 1.25 mL/min. Temperature programming was from 50 to 220 °C at 2 °C/min, and the temperature of the injector and transfer lines was 250 °C. Split flow was 20 ml/min (split ratio 1:16) and injection volume was 0.4 µL. All MS analyses were made in the electron impact (EI+) mode at 70 eV, the mass range was from 40 to 650 *m*/*z*, and the chromatogram acquired in total ion current (TIC). Compound identification was based on matching the MS spectrum with the NIST14 library and with a self-built EOs/terpenes library, then confirming the identity with Kovat’s type retention index [29]. Determination of retention indexes was achieved by injecting in GC-MS under identical settings as the essential oil to a reference mixture of linear alkanes (C6-C32) and processing results with Equation (9), where n and N are the number of carbons the linear alkanes were eluting, respectively, just before and just after the unknown compound and the rt’ for each compound (unknown or reference alkane) was the measured retention time (rt) corrected by subtracting the rt of an unretained reference.

(9) $$Kovat^{\prime }s\;Index=100\left[n+\left(N-n\right)\frac{\log{\mathrm{rt}}^{\prime }\left(unknown\right)-\log{\mathrm{rt}}^{\prime }\left(n\right)}{\log{\mathrm{rt}}^{\prime }\left(N\right)-\log{\mathrm{rt}}^{\prime }\left(n\right)}\right]$$

Semi-quantitative analysis of each oil was based on the relative % area of the chromatographic peak over the total area in the chromatogram, by means of 3 repeated injections in GC-FID, under identical settings used for identification in GC-MS. These values were also used to estimate the mass ratio (e.g., the relative concentration in g/L) among the components, assuming an RRF of 1 for all components.

### 3.3. Inhibited Autoxidation Studies

The absolute reactivity of EOs with peroxyl radicals was determined from their kinetics of oxygen consumption during the inhibited autoxidation of cumene or squalene. The reaction was thermally initiated at constant rate (*R*_i_ in the range (2–9) × 10^−9^ Ms^−1^) by the decomposition of AIBN (1–5) × 10^−2^ M), and the oxygen consumption was measured in a differential oxygen-uptake apparatus developed in our laboratory, based on a Validyne DP15 pressure transducer (Northridge, CA, USA), which has been previously described [45]. Tocopherol’s mimic 2,2,5,7,8-pentamethyl-6-chromanol (PMHC) was used as a reference antioxidant [46,47]. In a typical experiment, an air-saturated solution of the oxidizable substrate (50% *v*/*v*, corresponding to 3.6 M and 1.04 M for cumene and squalene, respectively) containing AIBN (0.05 M) in PhCl (sample) is equilibrated at 30 °C with an identical reference solution containing an excess of PMHC (25 mM) so as to block any radical chain in the reference and avoid significant consumption of the antioxidant therein during the experiment. After reaching a constant O_2_ consumption in the sample, a stock solution of the antioxidant in PhCl was injected in the sample flask. From the slope of the oxygen consumption during the inhibited period, *k*_inh_ values were obtained using Equation (7) [18,40,48,49,50], where n was the stoichiometric coefficient of the antioxidant set as *n* = 2 and *k*_p_ was the rate constant for chain propagation of the substrate: *k*_p_ (in PhCl, 30 °C) was 0.34 M^−1^s^−1^ for cumene [16] and 68 M^−1^s^−1^ for squalene [40]. The length of the inhibition period (τ) was determined graphically as the crosspoint between the initial tangent of the inhibited period and the final tangent of the not-inhibited period; however, in critical cases like curve **e** in Figure 2a, a valuable alternative was offered by the integral method described by Loshadkin et al. [51]. In the autoxidations of squalene, where there was no clear inhibited period or the experiment was stopped before its conclusion, kinetic analysis was performed using Equation (10), where *R*_0_ and *R*_AH_ were the rate of oxygen consumption in the absence or presence of the antioxidant, and 2k_t_ was the bimolecular termination rate constant (Equation (4)) of cumene or squalene [15,49,50]. Values of 2*k*_t_ (in PhCl, 30 °C) were 4.5 × 10^4^ M^−1^s^−1^ for cumene [16] and 7.4 × 10^6^ M^−1^s^−1^ for squalene [40]. Measurements were performed at three different concentrations for each EO: 1 mg/L, 100 mg/L and an intermediate concentration in the range 30–50 mg/L (see Figure S6) to check the validity of Equation (10). Although Equation (10) would best be used by plotting (R_0_/*R*_AH_ − *R*_AH_/*R*_0_) vs. [AH] and determining *k*_inh_ from its slope [49,50], data obtained at 1 mg/L were discarded form the analysis, as no sizeable inhibition was observed. Hence, the oxygen uptake plots at the remaining two concentrations were directly processed by Equation (10) and the results were averaged. The *R*_i_ value for any experimental settings was determined in preliminary experiments using PMHC as a reference antioxidant by means of Equation (8).

(10) $$\frac{R_{0}}{R_{\mathrm{AH}}}-\frac{R_{\mathrm{AH}}}{R_{0}}=\frac{nk_{\mathrm{inh}}{\left[\mathrm{AH}\right]}_{0}}{\sqrt{2k_{t}R_{i}}}$$

### 3.4. Statistical Analysis

Each measurement was performed in triplicate and results are expressed as mean ± SD (standard deviation).

## 4. Conclusions

The five tested EOs of red thyme, oregano, savory, clove bud, and cinnamon contained phenols as the main components: respectively, carvacrol and thymol for the first three and eugenol for the remaining two. Overall, the phenolic content represented 38 to 81% of the EO. Inhibited autoxidation studies of cumene and squalene indicated that all the tested EOs offered very good protection at a dose that depended on the oxidizability of the substrate. For cumene, full protection was achieved with 1 mg/L of the EO, while squalene required 100 mg/L for full protection, corresponding to 0.02% *w*/*v* on the basis of the oxidizable substrate. Kinetic analysis of the oxygen consumption traces matched with GC and GC-MS analysis of the EOs revealed that their antioxidant protection was nearly entirely due to the phenolic components and proportional to their content in the EO. Inhibition rate constants for trapping peroxyl radicals where measured for the first time for these Eos, revealing *k*_inh_ values in the order of 1.0 × 10^4^ M^−1^s^−1^ (30 °C) i.e., of the same magnitude of the widely used BHT, proving on unbiased quantitative grounds that all the tested EOs offer equivalent protection and can replace this synthetic antioxidant, particularly in high-end applications like food products, cosmetics, and pharmaceuticals. These findings call for further studies on a broader range of essential oils.

## Figures and Tables

**Table 1:** Composition of the investigated EOs, identified by CG-MS analysis. Concentrations are expressed as % peak area (±SD, *n* = 3) in the chromatogram from GC-FID analysis.

EO	*d* (g/mL, 20 °C)	Component	% (p/p)
*T. vulgaris*, L. (thyme)	0.912	α-Thujene	1.6 ± 0.1
		α-Pinene	3.8 ± 0.1
		Camphene	8.6 ± 0.2
		β-Pinene	2.6 ± 0.1
		p-Cymene	45.3 ± 1.2
		Thymol	4.0 ± 0.1
		Carvacrol	33.9 ± 0.7
*O. vulgare*, L. (oregano)	0.948	α-Phellandrene	0.4 ± 0.0_2_
		α-Pinene	0.7 ± 0.1
		Myrcene	1.2 ± 0.1
		α-Terpinene	1.1 ± 0.1
		p-Cymene	14.1 ± 0.3
		γ-Terpinene	4.9 ± 0.1
		Linalool	2.1 ± 0.1
		Thymol	0.4 ± 0.0_3_
		Carvacrol	69.2 ± 1.4
		β-Caryophyllene	1.6 ± 0.2
*S. hortensis*, L. (savory)	0.937	α-Pinene	1.8 ± 0.1
		Camphene	0.9 ± 0.1
		β-Pinene	0.2 ± 0.0_2_
		Myrcene	1.3 ± 0.1
		α-Terpinene	2.3 ± 0.1
		p-Cymene	20.0 ± 0.8
		Limonene	0.9 ± 0.0_4_
		Eucalyptol	0.6 ± 0.0_2_
		γ-Terpinene	17.0 ± 0.6
		Thymol	1.7 ± 0.1
		Carvacrol	46.6 ± 1.7
		Thymol Acetate	0.6 ± 0.0_2_
		β-Caryophyllene	1.6 ± 0.1
		Aromadendrene	0.9 ± 0.0_3_
		δ-Cadinene	0.3 ± 0.0_2_
		Caryophyllene Oxide	0.4 ± 0.0_2_
*E. caryophyllus*, Spreng (clove bud)	1.041	Eugenol	80.8 ± 1.7
		β-Caryophyllene	8.9 ± 0.5
		Humulene	1.1 ± 0.2
		Eugenyl Acetate	9.1 ± 0.6
*C. zeylanicum*, Blume (cinnamon)	1.043	α-Pinene	2.7 ± 0.1
		Camphene	0.8 ± 0.0_3_
		β-Pinene	0.4 ± 0.0_2_
		α-Phellandrene	1.7 ± 0.1
		p-Cymene	1.9 ± 0.1
		Linalool	2.1 ± 0.2
		Eugenol	81.4 ± 1.6
		β-Caryophyllene	4.8 ± 0.3
		Eugenyl Acetate	3.7 ± 0.1
		Caryophyllene Oxide	0.2 ± 0.0_4_
		Benzyl Benzoate	0.3 ± 0.0_4_

**Table 2:** Inhibition rate constants at 30 °C (PhCl) measured for the EOs in the autoxidation of cumene and squalene and apparent concentration of the antioxidant (mean ± SD), matched to the concentration of phenolics estimated from analysis of the EO (1 mg/L).

EO	Phenol	[Phenol]/M ^1^	Σ[Phenol]/M ^2^	[AH]_app_/M ^3^	*k*_inh_/M^−1^s^−1^(cumene) ^4^	*k*_inh_/M^−1^s^−1^(squalene) ^5^
*T. vulgaris*, L.	carvacrol	2.3 × 10^−6^	2.6 × 10^−6^	(2.5 ± 0.1) × 10^−6^	(1.5 ± 0.1) × 10^4^	(1.0 ± 0.3) × 10^4^
	thymol	2.7 × 10^−7^				
*S. hortensis*, L.	carvacrol	3.1 × 10^−6^	3.2 × 10^−6^	(2.9 ± 0.3) × 10^−6^	(1.3 ± 0.1) × 10^4^	(9.8 ± 1.5) × 10^3^
	thymol	1.1 × 10^−7^				
*O. vulgare*, L.	carvacrol	4.6 × 10^−6^	4.6 × 10^−6^	(4.8 ± 0.2) × 10^−6^	(1.3 ± 0.2) × 10^4^	(9.5 ± 0.9) × 10^3^
	thymol	0.3 × 10^−7^				
*E. caryophyllus*, Spreng	eugenol	4.9 × 10^−6^	4.9 × 10^−6^	(4.6 ± 0.3) × 10^−6^	(5.5 ± 0.5) × 10^3^	(5.7 ± 0.6) × 10^3^
*C. zeylanicum*, Blume	eugenol	5.0 × 10^−6^	5.0 × 10^−^^6^	(4.4 ± 0.4) × 10^−^^6^	(4.9 ± 0.3) × 10^3^	(4.8 ± 0.4) × 10^3^

^1^ Estimated from a GC-FID analysis of the EO added to the autoxidizing mixture at 1 mg/L. ^2^ Sum of phenolic components from the EO in the autoxidizing mixture. ^3^ Apparent effective concentration of the antioxidant in the mixture of cumene autoxidation, determined from the length of the inhibited period (Equation (8); [AH]_app_ = [AH_0_]). ^4^ Measured in the inhibited autoxidation of cumene. ^5^ Measured in the inhibited autoxidation of squalene.

## Data Availability

The data presented in this study are available on request from the corresponding author.

## Supplementary Materials

The following are available online, Figure S1–S5: chromatograms of the essential oils, Tables S1–S5: Tables of compound identification via GC-MS and Kovat’s Index, Figure S6: an example of raw oxygen uptake plots in squalene inhibition by oregano EO.

## Sample Availability

All compounds are commercially available.
