# Supplementary material for: Absolute Antioxidant Activity of Five Phenol-Rich Essential Oils
Source: Molecules. 2021 Aug 29;26(17):5237. doi: 10.3390/molecules26175237 (PMC8434318; doi:10.3390/molecules26175237)
Supplement: Supplementary file 1 [file molecules-26-05237-s001.zip › molecules-1352512-supplementary.pdf]

molecules

MDPI

Absolute antioxidant activity of five phenols-rich essential oils

Yafang Guo [1], Romeo Pizzol [1], Simone Gabbanini [2], Andrea Baschieri [3], Riccardo Amorati [1] and Luca Valgimigli [1,*]

[1] University of Bologna, Department of Chemistry "G. Ciamician", Via S. Giacomo 11 - 40126 Bologna, Italy.
[2] BeC s.r.l. - Research & Development, Via C. Monteverdi 49 – 47122 Forlì, Italy. laboratorio@bec-natura.com.
[3] ISOF – CNR, Consiglio Nazionale delle Ricerche, Via P. Gobetti 101 - 40129 Bologna, Italy.
* Correspondence: luca.valgimigli@unibo.it; Tel.: +39 051 2095683.

SUPPLEMENTARY MATERIAL

Table of contents

**Table S1**. Result of GC-MS analysis of *Thymus vulgaris,* L. (red thyme) essential oil on ZB-5 column.	p. 2
**Figure S1**. TIC chromatogram of *Thymus vulgaris,* L. (red thyme) essential oil obtained by GC-MS	p. 3
**Table S2**. Result of GC-MS analysis of *Origanum vulgare,* L. (oregano) essential oil on ZB-5 column	p. 4
**Figure S2**. TIC chromatogram of *Origanum vulgare,* L. (oregano) essential oil obtained by GC-MS	p. 5
**Table S3**. Result of GC-MS analysis of *Satureja hortensis,* L. (savory) essential oil on ZB-5 column	p. 6
**Figure S3**. TIC chromatogram of *Satureja hortensis,* L. (savory) essential oil obtained by GC-MS	p. 7
**Table S4**. Result of GC-MS analysis of *Eugenia caryophyllus*, Spreng (or *Syzygium aromaticum*, L, clove bud.) essential oil on ZB-5 column	p. 8
**Figure S4**. TIC chromatogram of of *Eugenia caryophyllus*, Spreng (or *Syzygium aromaticum*, L, clove bud.) essential oil obtained by GC-MS	p. 8
**Table S5**. Result of GC-MS analysis of *Cinnamomum zeylanicum,* Blume (cinnamon) essential oil on ZB-5 column	p. 9
**Figure S5**. TIC chromatogram of *Cinnamomum zeylanicum,* Blume (cinnamon) essential oil obtained by GC-MS	p. 10
**Figure S6.** Example of one set or raw kinetic oxygen uptake plots for the inhibition of squalene autoxidation by oregano EO.	p. 11

Table S1. Result of GC-MS analysis of *Thymus vulgaris* L. (red thyme) essential oil on ZB-5 column (30m×0.25mm×0.25µm) from 50 to 220°C at 2°C/min. Identification of peaks is obtained with Kovat's Index and fit to NIST14 library (retro-Fit % accuracy is shown in the table).

Peak #	rt [min]	Nist / Kovat's ID	Area %	NIST'14 r-Fit %	Kovat's Index found	Kovat's Index tabulated	Δ Kovats
1	5,07	α-Thujene	7,01	92,1	922	931	9
2	5,41	α-Pinene	4,02	95,8	934	937	3
3	5,92	Camphene	8,40	97,1	950	952	2
4	6,89	β-Pinene	2,61	95,7	976	979	3
5	8,87	p-Cymene	42,03	99,0	1024	1025	1
6	24,38	Thymol	3,83	96,1	1289	1291	2
7	24,82	Carvacrol	32,10	95,2	1295	1296	1

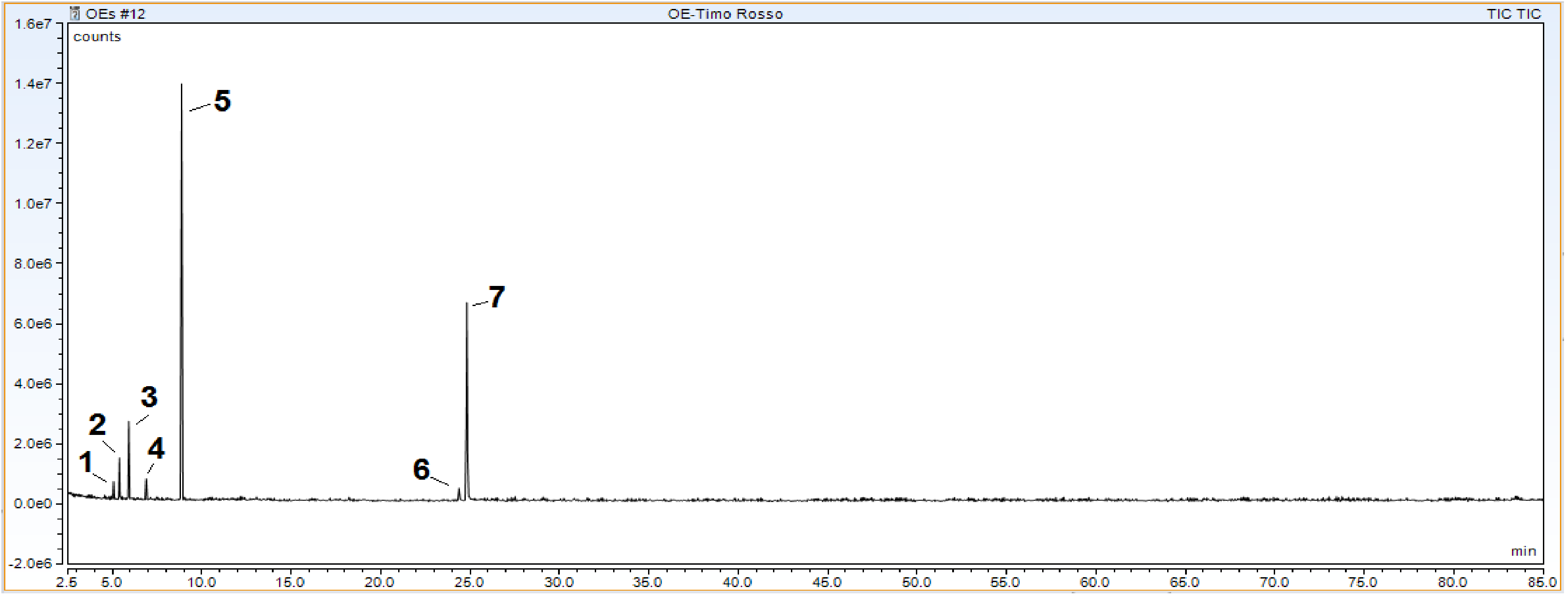

Figure S1. TIC chromatogram of *Thymus vulgaris*, L. (red thyme) essential oil obtained by GC-MS on ZB-5 column (30m×0.25mm×0.25μm) from 50 to 220°C at 2°C/min. Peak number correspond to Table S1.

Table S2. Result of GC-MS analysis of *Origanum vulgare*, L. (oregano) essential oil on ZB-5 column (30m×0.25mm×0.25μm) from 50 to 220°C at 2°C/min. Identification of peaks is obtained with Kovat's Index and fit to NIST14 library (retro-Fit % accuracy is shown in the table).

Peak #	rt [min]	Nist / Kovat's ID	Area %	NIST'14 r-Fit %	Kovat's Index found	Kovat's Index tabulated	Δ Kovats
1	5,25	α-Phellandrene	0,46	95,8	929	929	0
2	5,41	α-Pinene	0,76	97,9	934	937	3
3	7,53	Myrcene	1,30	97,9	991	991	0
4	8,61	α-Terpinene	1,18	97,6	1017	1016	-1
5	8,87	p-Cymene	14,50	99,5	1024	1025	1
6	10,54	γ-Terpinene	5,80	99,1	1059	1060	1
7	12,76	Linalool	2,26	97,6	1098	1099	1
8	18,14	α-Terpineol	0,55	95,4	1190	1190	0
9	24,38	Thymol	0,35	98,8	1289	1291	2
10	24,82	Carvacrol	68,21	99,0	1295	1296	1
11	31,65	β-Caryophyllene	1,95	98,5	1405	1406	1

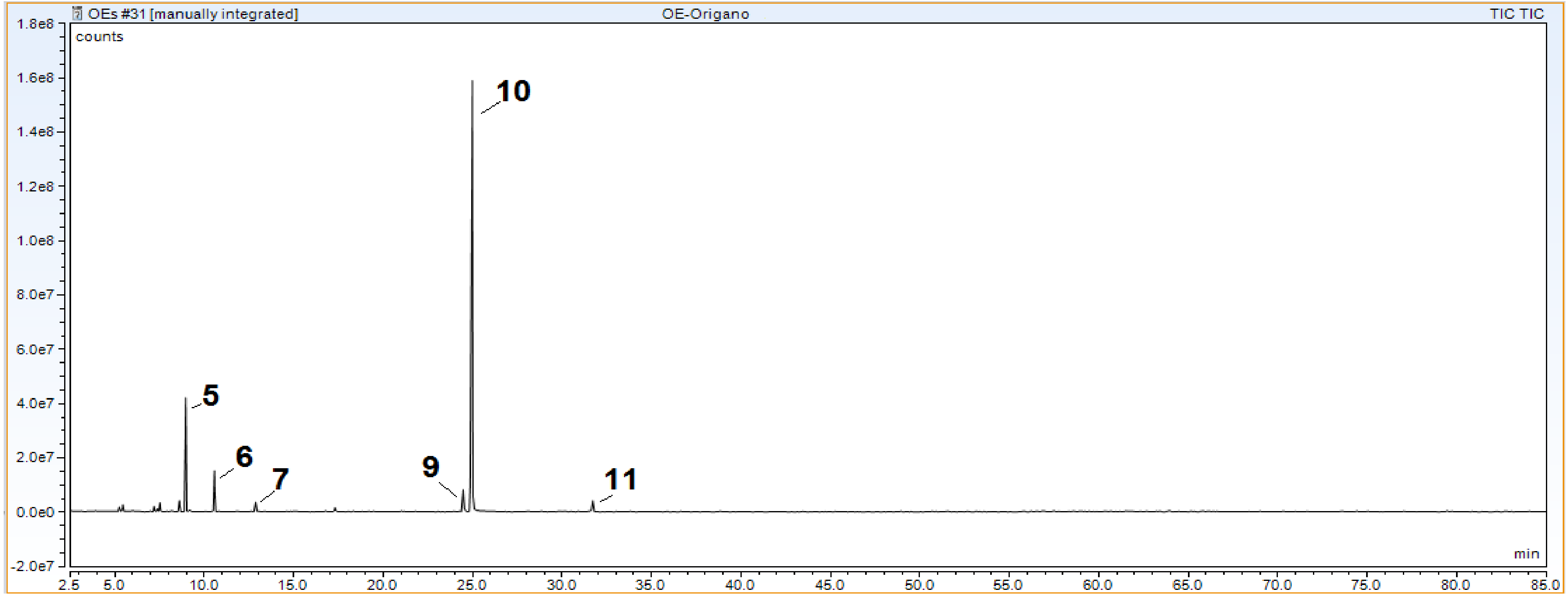

Figure S2. TIC chromatogram of *Origanum vulgare,* L. (oregano) essential oil obtained by GC-MS on ZB-5 column (30m×0.25mm×0.25µm) from 50 to 220°C at 2°C/min. Peak number correspond to Table S2.

Table S3. Result of GC-MS analysis of *Satureja hortensis*, L. (savory) essential oil on ZB-5 column (30m×0.25mm×0.25µm) from 50 to 220°C at 2°C/min. Identification of peaks is obtained with Kovat's Index and fit to NIST14 library (retro-Fit % accuracy is shown in the table).

Peak #	rt [min]	Nist / Kovat's ID	Area %	NIST'14 r-Fit %	Kovat's Index found	Kovat's Index tabulated	Δ Kovats
1	5,41	α-Pinene	2,20	99	934	937	3
2	5,92	Camphene	0,91	91,2	950	952	2
3	6,89	β-Pinene	0,20	97,6	976	978	2
4	7,53	Myrcene	1,42	98,5	991	991	0
5	8,61	α-Terpinene	2,43	98,2	1017	1016	-1
6	8,87	p-Cymene	19,40	99,5	1024	1025	1
7	9,15	Limonene	1,11	94,5	1030	1030	0
8	9,19	Eucalyptol	0,80	93,1	1031	1032	1
9	10,54	γ-Terpinene	17,91	98,8	1059	1060	1
10	24,38	Thymol	1,42	98,6	1289	1291	2
11	24,82	Carvacrol	45,80	98,7	1295	1296	1
12	27,69	Thymol Acetate	0,82	97,9	1343	1338	-5
13	31,65	β-Caryophyllene	1,73	95,4	1405	1406	1
14	37,42	Aromadendrene	1,22	90,1	1499	1459	-40
15	37,87	δ-Cadinene	0,41	96,5	1508	1508	0
16	41,23	Caryophyllene Oxide	0,60	93,3	1566	1574	8

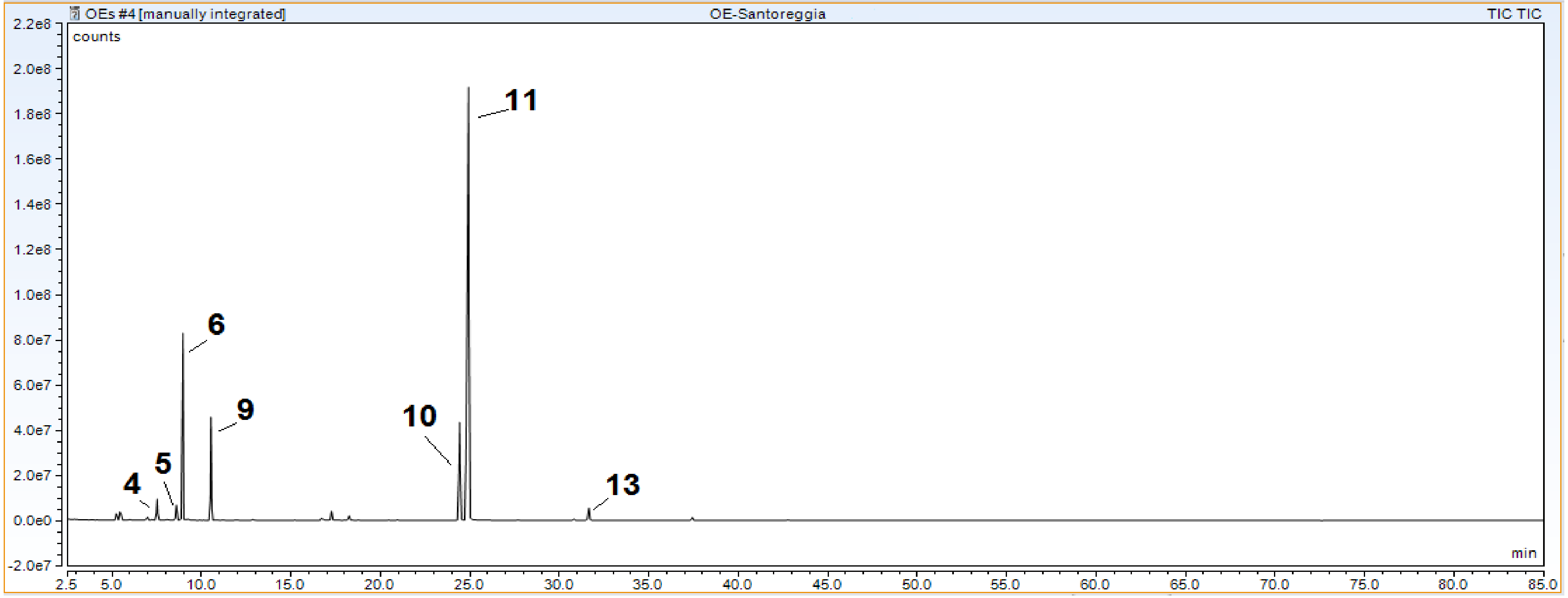

Figure S3. TIC chromatogram of *Satureja hortensis,* L. (savory) essential oil obtained by GC-MS on ZB-5 column (30m×0.25mm×0.25µm) from 50 to 220°C at 2°C/min. Peak number correspond to Table S3.

Table S4. Result of GC-MS analysis of *Eugenia caryophyllus*, Spreng (or *Syzygium aromaticum*, L, clove bud.) essential oil on ZB-5 column (30m×0.25mm×0.25µm) from 50 to 220°C at 2°C/min. Identification of peaks is obtained with Kovat's Index and fit to NIST14 library (retro-Fit % accuracy is shown in the table).

Peak #	rt [min]	Nist / Kovat's ID	Area %	NIST'14 r-Fit %	Kovat's Index found	Kovat's Index tabulated	Δ Kovats
1	27,96	Eugenol	80,41	95,8	1348	1348	0
2	31,72	β-Caryophyllene	8,60	96,4	1406	1404	-2
3	33,85	Humulene	0,82	94,4	1443	1454	11
4	38,08	Eugenyl Acetate	10,10	98,6	1511	1524	13

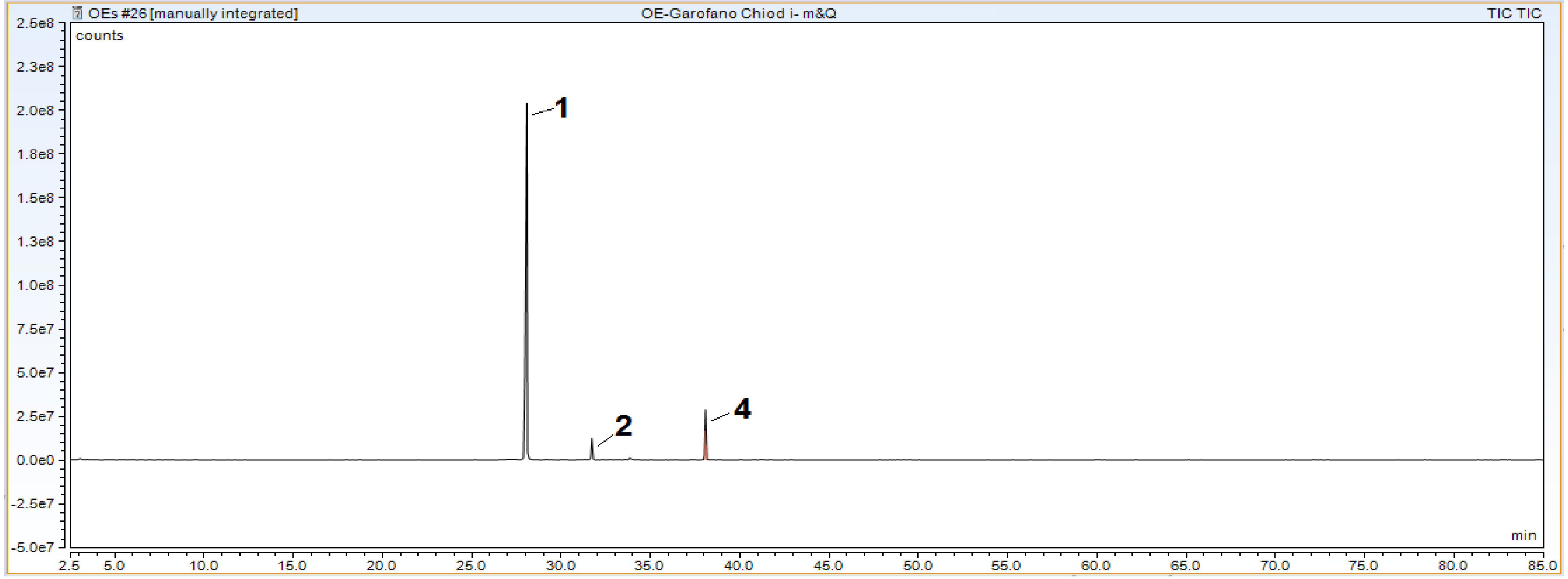

Figure S4. TIC chromatogram of of *Eugenia caryophyllus*, Spreng (or *Syzygium aromaticum*, L, clove bud.) essential oil obtained by GC-MS on ZB-5 column (30m×0.25mm×0.25µm) from 50 to 220°C at 2°C/min. Peak number correspond to Table S4.

Table S5. Result of GC-MS analysis of *Cinnamomum zeylanicum*, Blume (cinnamon) essential oil on ZB-5 column (30m×0.25mm×0.25μm) from 50 to 220°C at 2°C/min. Identification of peaks is obtained with Kovat's Index and fit to NIST14 library (retro-Fit % accuracy is shown in the table).

Peak #	rt [min]	Nist / Kovat's ID	Area %	NIST'14 r-Fit %	Kovat's Index found	Kovat's Index tabulated	Δ Kovats
1	5,41	α-Pinene	2,80	98,6	934	937	3
2	5,92	Camphene	0,91	96,7	950	952	2
3	6,89	β-Pinene	0,50	94,6	976	978	2
4	8,14	α-Phellandrene	1,41	95,8	1006	1005	-1
5	8,87	p-Cymene	1,80	98,2	1024	1025	1
6	12,76	Linalool	2,62	96,1	1098	1099	1
7	27,96	Eugenol	80,60	99,4	1348	1348	0
8	31,70	β-Caryophyllene	4,41	98,1	1405	1404	-1
9	38,08	Eugenyl Acetate	3,13	94,2	1511	1522	11
10	41,23	Caryophyllene Oxide	0,30	88,1	1566	1574	8
11	44,88	Benzyl Benzoate	1,51	97,9	1776	1765	-11

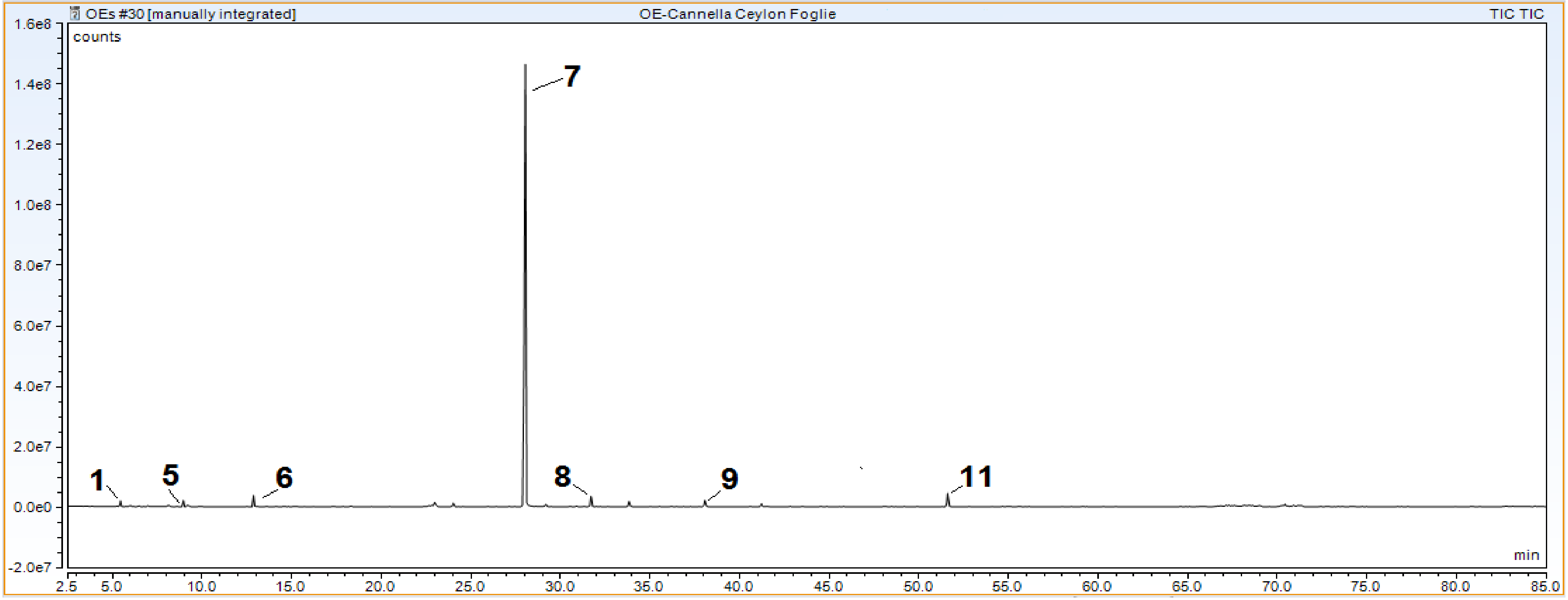

Figure S5. TIC chromatogram of *Cinnamomum zeylanicum,* Blume (cinnamon) essential oil obtained by GC-MS on ZB-5 column (30m×0.25mm×0.25µm) from 50 to 220°C at 2°C/min. Peak number correspond to Table S5.

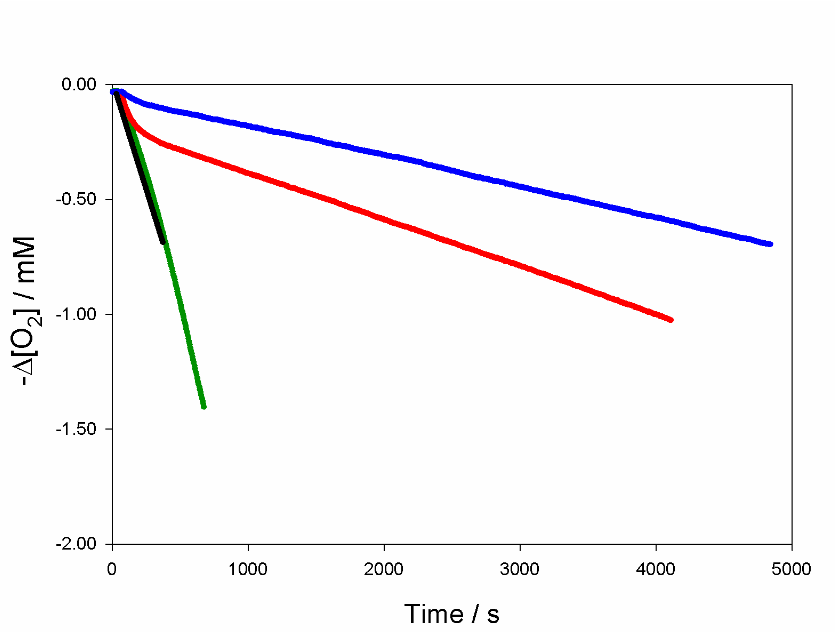

Figure S6. Example of one set or raw kinetic oxygen uptake plots for the inhibition of squalene autoxidation by *Origanum vulgare,* L. (oregano) EO. Plots represent the oxygen consumption during the autoxidation of squalene (1.04 M) initiated by AIBN (0.05 M) in PhCl at 30°C without inhibitors (black) or in the presence of oregano EO at different concetrations: 1 mg/L (green), 50 mg/L (red), or 100 mg/L (blue).
